# Incorporating Genome-Wide Association Mapping Results Into Genomic Prediction Models for Grain Yield and Yield Stability in CIMMYT Spring Bread Wheat

**DOI:** 10.3389/fpls.2020.00197

**Published:** 2020-03-04

**Authors:** Deepmala Sehgal, Umesh Rosyara, Suchismita Mondal, Ravi Singh, Jesse Poland, Susanne Dreisigacker

**Affiliations:** ^1^Global Wheat Program, International Maize and Wheat Improvement Center, Texcoco, Mexico; ^2^Department of Plant Pathology, Kansas State University, Manhattan, KS, United States

**Keywords:** *Triticum aestivum*, GBS, GWAS, haplotypes, genomic selection

## Abstract

Untangling the genetic architecture of grain yield (GY) and yield stability is an important determining factor to optimize genomics-assisted selection strategies in wheat. We conducted in-depth investigation on the above using a large set of advanced bread wheat lines (4,302), which were genotyped with genotyping-by-sequencing markers and phenotyped under contrasting (irrigated and stress) environments. Haplotypes-based genome-wide-association study (GWAS) identified 58 associations with GY and 15 with superiority index *Pi* (measure of stability). Sixteen associations with GY were “environment-specific” with two on chromosomes 3B and 6B with the large effects and 8 associations were consistent across environments and trials. For *Pi*, 8 associations were from chromosomes 4B and 7B, indicating ‘hot spot’ regions for stability. Epistatic interactions contributed to an additional 5–9% variation on average. We further explored whether integrating consistent and robust associations identified in GWAS as fixed effects in prediction models improves prediction accuracy. For GY, the model accounting for the haplotype-based GWAS loci as fixed effects led to up to 9–10% increase in prediction accuracy, whereas for *Pi* this approach did not provide any advantage. This is the first report of integrating genetic architecture of GY and yield stability into prediction models in wheat.

## Introduction

Bread wheat (*Triticum aestivum* L.) is one of the most important cereal crops for global food security (FAOSTAT, 2016). With the predicted detrimental effects of climate change on its production and the projected global demand by 2050, there is a pressing need to accelerate the development of high yielding varieties (Hellin et al., 2012). Improvement of grain yield (GY) therefore is a prime target for wheat breeders globally. GY is a complex trait governed by many loci of small-effects with significant loci × loci interactions (Arzani and Ashraf, 2017; Sehgal et al., 2017). In addition, strong genotype × environment interaction associated with GY makes its genetic improvement an arduous task.

Latest advances in sequencing technologies, providing millions of single nucleotide polymorphism (SNP) markers at a low cost, have revolutionized the field of plant genomics. Hence, a paradigm shift from marker-based to sequencing-based genotyping of breeders’ germplasm panels has been observed in the post-genome sequencing era. Wheat has particularly benefited from these technological advancements; dense sets of SNPs are now available from different marker platforms (90K Illumina iselect, genotyping by sequencing (GBS), DArTseq, high-density Affymetrix Axiom^®^ genotyping array). Due to the transformed genetic toolkit available in wheat, untangling the genetic architecture of traits by genome-wide association study (GWAS) and predicting performance by genomic selection (GS) have become feasible. Several GWAS analyses have been performed in wheat for plethora of traits including yield and yield components (Golabadi et al., 2011; Neumann et al., 2011; Bordes et al., 2014; Edae et al., 2014; Azadi et al., 2015; Assanga et al., 2017; Bhusal et al., 2017; Li et al., 2019). However, outcomes of these studies have hardly been applied in practical breeding programs.

Genomic selection is another widely used genomics-assisted approach in which genome-wide markers are used to predict the breeding value of individuals in a breeding population. It offers the potential to accelerate genetic gain by increasing selection accuracy and intensity and shortening the lengths of breeding cycles. Though GS is a relatively new technology for wheat breeding, significant success has been achieved in testing and validating various models for GY and other traits using markers and pedigrees (de los Campos et al., 2009; Crossa et al., 2010, 2011, 2016; Heffner et al., 2011; Burgueño et al., 2012; Pérez-Rodríguez et al., 2012; Rutkoski et al., 2014; Juliana et al., 2017a, b).

In animal breeding, evidences have accumulated to realize that integration of prior information of quantitative trait loci (QTL) in GS models can result in increased prediction accuracies for traits with complex genetic architecture (Boichard et al., 2012; Su et al., 2014; Zhang et al., 2014; Brøndum et al., 2015; Veroneze et al., 2016; Lopes et al., 2017). A comprehensive simulation study in plants suggested that by using a few (1–3) major genes/QTL as fixed effects in GS models, it might be possible to increase the accuracy of GS for quantitative traits (Bernardo, 2014), if each gene contributes to ≥10% of the variance. QTL with large effects (≥10%) have been identified for less complex traits in wheat (e.g., rust resistance) in bi-parental populations and have been integrated in GS models as fixed effects to improve prediction accuracies (Rutkoski et al., 2014). However, such large effect QTLs are rarely identified for complex traits such as GY in a typical GWAS study (Sehgal et al., 2016, 2017). The potential to integrate consistent and robust associations identified from GWAS as fixed variables in GS models to improve prediction accuracy for complex traits has not been investigated comprehensively in plants (Spindel et al., 2016; He et al., 2016).

In this study, we used a large set of spring bread wheat lines (4,302) from the CIMMYT Global Wheat Program (Supplementary Table S1), genotyped with GBS markers and phenotyped under multiple contrasting environments, to; (a) untangle the genetic architecture of GY and yield stability; (b) identify robust GY-QTLs for specific environments and GY-QTLs with consistent favorable allele across environments and trials; and (c) evaluate the importance of these QTL in improving genomic prediction accuracies by integrating them as fixed effects in GS models.

## Materials and Methods

### Plant Materials and Phenotyping

Plant materials consisted of 4,302 spring bread wheat lines, which formed the entries of five Elite Yield Trials (EYT) during five consecutive years (Supplementary Table S1) i.e., EYT2011-12, EYT2012-13, EYT2013-14, EYT2014-15, and EYT2015-16 and comprised 643, 905, 983, 942, and 829 lines, respectively. Each trial year the breeding program selects 1092 new advanced lines for 2nd year yield testing which is the source for the lines above. Each year the lines are different except for the checks. All EYTs were phenotyped at the Norman E. Borlaug experimental research station (CENEB) in Ciudad Obregon, Mexico. The 1092 lines in each year were divided into 39 experiments, each with 28 entries, 2 checks in an alpha lattice design with 3 replications. Small size units (30 plots with 28 entries and 2 checks) are used to minimize the field variation, which simplifies selection. Each year all 1092 lines were sown in five contrasting environments by modulating planting date and irrigation, including optimum and stressed environments, in combination with two management conditions of raised bed planting (B) or flat planting (F). The optimum environments included two well-irrigated treatments with five irrigations (5IR) under bed and flat planting (B-5IR and F-5IR).

The three stress environments included (i) mild drought stress; sown in bed and with only two irrigations (B-2IR), (ii) severe drought stress; sown in flat and with drip irrigation (SD) and (iii) heat stress; bed sowing with five irrigations (HS; average T_max_ > 32°C). All trials were sown in mid-November except for HS, which was sown in the end of February. The plot size in bed planting was 2.8 m × 1.6 m (two beds of 0.8 m with three rows each) and in flat planting was 4 m × 1.6m (six rows). Trials were phenotyped for days to heading (DH), plant height (PH), and GY in each year. DH was recorded as the number of days from planting until 50% of the spikes in each plot had completely emerged above the flag leaves. PH was recorded as the average of three values for each plot measured in centimeter from the soil surface to the tip of the spike excluding awns. At maturity whole plots were harvested to estimate GY per plot. The details of the phenotyping are also described in Sehgal et al. (2017).

### Statistical Analyses of Phenotypic Data

Each combination of EYT and simulated environment was defined as one trial, resulting in 25 trials (five EYTs × five environments) for each trait. The data were adjusted for block effects within each replication per trial using values from the two common check varieties in SAS 9.4 using the PROC GLM function and adjusted entry mean of the genotypes were calculated for GY.

For DH and PH, the adjusted means were calculated by the formula Y = (Y_*ij*_-Y_*i*_) + Y_all trials_, where Y_*ij*_ is value of the entry for a trial, Y_*i*_ is mean of checks of that trial and Y_all trials_ is the mean of checks of all trials.

The summary statistics function in GenStat edition 14th was used to obtain the minimum and maximum values of each trait in each trial. ANOVA was performed using a customized script in R. Two GY stability indices were calculated using GY data from five environments in each EYT; Lin and Binn’s superiority index (*Pi*; Lin and Binns, 1988) and Eberhart and Russell’s coefficient (ER; Eberhart and Russell, 1966).

### Genotyping and Haplotype Construction

All lines were genotyped using GBS at Kansas State University. GBS was conducted by 192-plexing on Illumina HiSeq2000 with 1 × 100 bp reads and subsequent SNP calling with TASSEL 5v2 pipeline as described in Rutkoski et al. (2016).

From an initial set of 20,794 SNP markers obtained on the 4,302 samples from five EYTs, a set of 8,443 filtered SNPs with a maximum 40% missing data and a minor allele frequency (MAF) ≥ 0.15 was used for constructing haplotype blocks. No further imputations were done with the filtered set of 8,443 SNPs. Since the haplotype blocks were created on all lines from five EYTs together, a high threshold for MAF i.e., MAF ≥ 0.15 was applied so that in each EYT a MAF ≥ 0.05 could be achieved. Haplotypes were generated based on the linkage disequilibrium (LD) parameter D’ using the modified R script from Gabriel et al. (2002) described in Sehgal et al. (2019). Briefly, we calculated D’ 95% confidence intervals between SNPs and categorized each comparison as ‘strong LD,’ ‘inconclusive,’ or ‘strong recombination.’ If 95% of the comparisons in one block were in ‘strong LD,’ a haplotype block was created. For two or more SNPs to be classified in ‘strong LD’, the minimum lower and upper confidence interval values were set to 0.6 and 0.95, respectively. The haplotype blocks were named as combinations of the prefix ‘HB’ for the haplotype block followed by a number, which represents the chromosome followed by a dot and incrementing number of the haplotype blocks along the chromosome. Two and three-locus interactions were studied using an in-house script executed in R as described in Sehgal et al. (2017). For single marker (SNP) GWAS, interactions were calculated for the associated 125 SNPs and among genome-wide SNPs. For haplotype-based GWAS, interactions were calculated for the associated 58 haplotype blocks and among genome-wide haplotype blocks. For both interaction analyses, *p* < 0.001 threshold was used as cutoff.

### Genome-Wide Association Mapping

Haplotype-based GWAS was conducted in each individual EYT using Plink version 1.07 (Purcell et al., 2007), while single marker-based GWAS was conducted in GAPIT V2 (Tang et al., 2016), both packages executed in R. The covariance matrix was derived by conducting principal component analysis (PCA) analysis using the function PRCOMP from the STATS package in R. The kinship matrix was calculated by the VanRaden algorithm (VanRaden, 2008). In both analysis (haplotype-based and single marker-GWAS), a mixed linear model was used with PCA as fixed variate and kinship as random. The appropriate number of principal components were assessed based on Bayesian information criterion (Schwarz, 1978). DH and PH were used as covariates to reduce confounding effects of phenological traits. In addition, major flowering genes present in high frequency in these EYTs i.e., *Ppd-D1a* and *Vrn-B1a* were also used as covariables to avoid any further confounding effects of flowering genes.

### Genomic Prediction Models

All genomic predictions were based on the G-BLUP model, using the following formula:

(1) y=X⁢β+Z⁢u+ϵ

where *y* is a vector of phenotypes consisting of the adjusted means, β is a vector of fixed effects (depending upon the model see the below), *u* is a vector of random genetic values, *e* is the vector of residuals. *X* and *Z* are design matrices. The *u* was assumed to follow a Gaussian distribution $u∼N\,\left(0,\text{G}\,\sigma_{\text{g}}^{2}\right)$, where **G** was the genomic relationship matrix and $\sigma_{\text{g}}^{2}$ was the additive genetic variance. The residuals *e* was assumed to follow a Gaussian normal distribution $u∼N\,\left(0,\text{I}\,\sigma_{\text{e}}^{2}\right)$, where I was the identity matrix.

In order to include the GWAS results in genomic prediction, four types of relationship matrices were calculated and used as part of the G-BLUP models in BGLR package:

(a) The additive relationship matrix (**G_M_**) was calculated using single markers and following the formula:

(2) $${\text{G}}_{M}={\text{MM}}^{T}$$

where ***M*** ∈ {1,0,−1} depending upon whether a particular marker carried the homozygous reference, heterozygous or homozygous alternate allele.

(b) The haplotype based relationship matrix (**G_H_**) was calculated using the following formula:

(3) $${\text{G}}_{H}={\text{HH}}^{T}$$

where ***H*** ∈ {1,0} depending upon whether particular haplotype allele was present or absent.

(c) The single marker-based Gaussian Kernel (**G_MG_**) – Marker based Gaussian Kernel was

(4) $${\text{G}}_{i\,j}=\text{exp}\,\left[-\left(D_{i\,j}\,/\,\theta \right)\right]$$

where

(5) $$D_{i\,j}={\left[\left(\frac{1}{4}\,\text{m}\right)\,\sum_{k=1}^{m}{\left(S_{i\,k}-S_{j\,k}\right)}^{2}\right]}^{1/2}$$

**Table:** The summary of the four models run is presented in the following table:

Model	Fixed effects	Relationship matrix	Description
1	Environment	G_M_	Base model
2	Environment	G_H_	501 haplotypes + Epistasis
3	Environment, predefined markers	G_MG_	8,443 single markers + GWAS markers + + Epistasis
4	Environment, predefined Haplotypes	G_HG_	501 haplotypes + GWAS haplotypes + + Epistasis

here θ is scale parameter, m = number of markers, **D**_*ij*_ is Euclidian distance calculated between individuals i and j using the marker scores S, normalized to the interval [0,1].

(d) The haplotype-based Gaussian Kernel (**G_*HG*_**)

In a similar way to **G**_*M*_, the haplotype based Gaussian Kernel based matrix was calculated except m is number of haplotypes and **S** is haplotype score indicating presence (1) and absence (0) for particular haplotype allele. Epistatis was captured in the models using Reproducing Kernel Hilbert Space (RKHS) regression equation based on Gaussian kernel (Yong and Jochen, 2015).

### Cross Validation

Cross validations were performed by randomly splitting of observations into 90% training set and 10% test set within each EYT. The prediction accuracy was calculated as correlation between true and predicted values in 10% test set. The cross validation was repeated 100 times to calculate mean and standard deviation of the prediction accuracy.

## Results

### Grain Yield Performances in Contrasting Environments

GGE plots of all environments showed that they were significantly different in each EYT with both principal components explaining 80.2, 81.7, 86.3, 84.7, and 88.9% of the overall variation in EYT2011-12, EYT2012-13, EYT2013-14, EYT2014-15, and EYT2015-16, respectively (Supplementary Figure S1). Mean GY across all trials and environments ranged from 1.62 t/ha (EYT2015-16 in SD) to 8.64 t/ha (EYT2011-12 in B-5IR) (Table 1). Means across the five EYTs for each environment accounted for 7.0, 6.7, 3.9, 2.5, and 3.5 t/ha for B-5IR, F-5IR, B-2IR, SD and HS, respectively. The percent reduction in GY under stress environments ranged from 18.5 to 56.4%, 50.1 to 77.1%, and 33.1 to 62.3% across EYTs under B-2IR, SD and HS, respectively (Supplementary Figure S2). SD was overall the lowest yielding environment, followed by HS. The differences in GY between irrigated environments (B-5IR and F-5IR) ranged from 0.72% in EYT2013-14 to 10.9% in EYT2012-13.

**TABLE 1:** Adjusted mean of GY, ANOVA and heritability (*h*^2^) of the five elite yield trials (EYT).

	**Mean GY (kg/ha)**									
	B-5IR	F-5IR	B-2IR	*SD*	HS		ANOVA			*h*^2^ (across envs.)
							**Df**	***F*-value**	**Pr (> F)**	
EYT2011-12	8622 ± 631	7892 ± 597	3763 ± 834	2662 ± 532	4275 ± 605	Rep	2	2.65	*	0.63
						Geno	642	8.93	***	
						Env	4	57129.90	***	
						Geno:Env	2568	4.82	***	
						Residuals	6426			
EYT2012-13	7737 ± 450	6879 ± 512	4741 ± 403	3438 ± 507	3576 ± 590	Rep	2	2.21	ns	0.32
						Geno	904	7.96	**	
						Env	4	84908.52	**	
						Geno:Env	3616	5.41	**	
						Residuals	9045			
EYT2013-14	6105 ± 512	6061 ± 672	3698 ± 388	2107 ± 529	2305 ± 504	Rep	2	15.91	**	0.51
						Geno	982	8.58	**	
						Env	4	71944.28	**	
						Geno:Env	3927	4.20	**	
						Residuals	9819			
EYT2014-15	5581 ± 493	5714 ± 541	4541 ± 369	2797 ± 704	3735 ± 641	Rep	2	10.54	**	0.57
						Geno	941	14.23	**	
						Env	4	35614.11	**	
						Geno:Env	3764	6.20	**	
						Residuals	9400			
EYT2015-16	7096 ± 365	7018 ± 605	3201 ± 392	1622 ± 556	3628 ± 443	Rep	2	7.51	**	0.27
						Geno	828	7.04	**	
						Env	4	118590.00	**	
						Geno:Env	3311	5.12	**	
						Residuals	8216			

Broad sense heritability for GY across environments ranged from 0.27 to 0.63, being highest in EYT2011-12 and lowest in EYT2015-16 (Table 1). Phenotypic correlations between days to heading (DH) and GYs were positive for the two irrigated environments and negative for all stress environments. Correlations of GY with plant height (PH) were positive irrespective of the environment (Supplementary Table S2). Supplementary Figures S3, S4 show G × E plots for *Pi* vs. mean GY in EYTs and correlations between mean GY and the two stability indices, respectively.

### Haplotype Analysis, Haplotype-Based GWAS and Its Comparison With Single Marker GWAS

Genome-wide, 501 haplotype blocks were constructed with a range from two to nine SNPs per block (Supplementary Table S3). A total of 4,038 SNPs built the 501 haplotype blocks. As expected, the wheat D sub-genome showed the lowest number of haplotype blocks (36) with the lowest number of haplotypes (78). Sub-genomes A and B showed 197 and 268 haplotype blocks and 506 and 656 haplotypes, respectively (Supplementary Figure S5).

**FIGURE 1:**
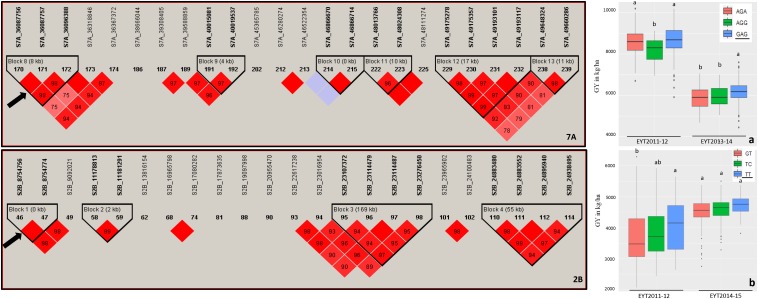
Partial haplotype maps of chromosomes 7A and 2B showing haplotype blocks HB19.8 and HB5.1 (indicated with an arrow) associated with GY in B-5IR and B-2IR environments, respectively (left part of figure). The numbers inside the diamonds are the *r*^2^ values between SNPs on a scale of 0 to 100%. Allelic effects of the haplotypes in blocks HB19.8 **(a)** and HB5.1 **(b)** on grain yield (*Y* axis in kg/ha) are shown on the right. The favorable haplotypes are underscored based on the highest mean.

**FIGURE 2:**
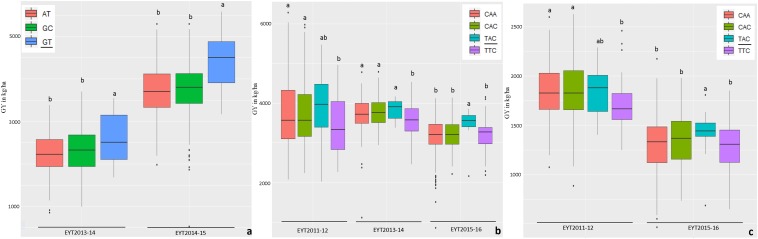
Allelic effects of haplotypes in HB8.26 (chromosome 3B) for GY (Y axis in kg/ha) under HS **(a)** and HB17.1 (chromosome 6B) under B-2IR **(b)** and SD **(c)** environments. The favorable alleles are underscored based on the highest mean.

Haplotype-based GWAS identified 58 haplotype blocks associated with GY across all 25 trials, ranging from 17 in EYT2012-13 to 33 in EYT2011-12 (Supplementary Table S4). The associated haplotype blocks could be divided into four groups: (1) associated with GY only in one EYT, (2) strictly associated with GY in a single environment across EYTs (3), consistently associated with GY across EYTs and multiple environments in each EYT (4), consistently associated with GY across EYTs but in varying environments (one to many). Out of the 58 haplotype-GY associations, 32 associations were categorized into groups 1 and 2 (16 each), the remaining 26 associations were assigned to groups 3 (8 associations) and 4 (18 associations). Figure 1 shows partial haplotype maps of chromosomes 7A and 2B showing allelic effects of two haplotypes blocks (HB19.8 and HB5.1) associated with GY assigned to group 2. The favorable haplotypes explained more than 5% increase in GY in two EYTs. The favorable allele ‘GT’ of another haplotype block (HB8.26) assigned to group 2 and identified in HS showed a 8.1 to 12.2% (189 – 374 kg/h) increase in GY across EYTs (Figure 2a). The haplotype block HB17.1 was assigned to group 3. The favorable allele ‘TAC’ of this block showed a 8–14% (240–530 kg/h) and 11.6–16.3% (244–303 kg/h) increase in GY across three EYTs, in B-2IR and SD respectively (Figures 2b,c).

Single marker-based GWAS obtained 125 SNP-GY associations for all 25 trials. Similar to the haplotype-GY associations, the associations in this analysis were divided into four groups. Most associations (91) were identified in only one EYT, belonging to the previously defined group 1. Of the remaining associations, 19, 8, and 7 were assigned to groups 2, 3, and 4, respectively (Supplementary Table S5). Twenty-nine SNPs significant in the single marker-based GWAS were also part of the 58 haplotype blocks associated with GY in the haplotype-based GWAS (Supplementary Table S6). For the SD and HS environments, three chromosome regions were identified by both analyses, but different markers were significantly associated with GY in each GWAS (Supplementary Table S7).

Two yield stability indices (*Pi* and ER) for GY were calculated. Of the two, Lin and Binns’s *Pi* is a compound index that quantifies both performance (G) and G × E interaction and thus identifies good performing and stable lines with a single parameter (Supplementary Figure S3). *Pi* index also showed a higher and positive correlation with mean GY than ER (Supplementary Figure S4). GWAS was only conducted for *Pi*. In the haplotype-based GWAS, 15 haplotype blocks were associated with *Pi*. Five of the haplotype blocks were identified in three EYTs and 10 haplotype blocks in two EYTs (Supplementary Table S8). In single marker-based GWAS for *Pi*, 28 SNPs were identified to be associated with *Pi* in two or three EYTs. Of these, 17 SNPs were part of the significantly associated haplotype blocks in the haplotype-based GWAS for *Pi* (Supplementary Table S8). The remaining 11 SNPs were identified on the same chromosome regions close to the associated haplotype blocks.

### Effects of Haplotypes and Single SNP Markers on GY and Pi

The percentage variation (*R*^2^) explained by 58 haplotypes associated with GY ranged from 0.7 to 14.0% in different environments across EYTs, while for the associated 125 SNPs it ranged from 0.02 to 4.5%. Similarly, for *Pi, R*^2^ ranged from 4.7 to 11.0% and 2.5 to 5.2% for haplotypes and single SNPs, respectively. The average *R*^2^ explained was 6.1–9.9% higher with the haplotype-based GWAS as compared to the single marker-based GWAS for GY and *Pi* across all EYTs (Supplementary Figure S6).

**FIGURE 3:**
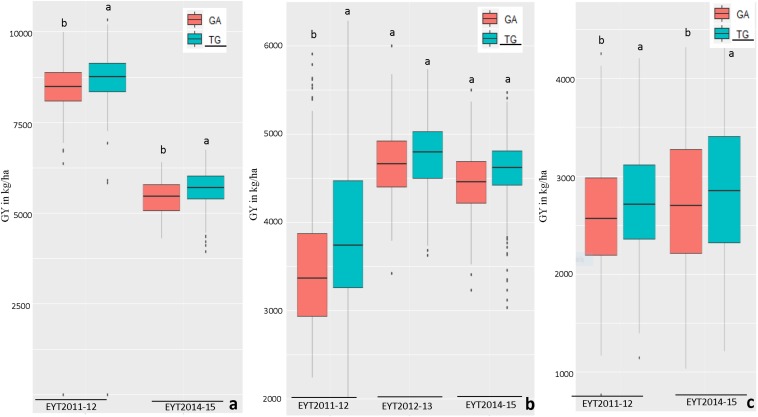
Allelic effects of two haplotypes in HB11.4 (associated with *Pi*) on GY (Y axis in kg/ha) across EYTs; TG showing the highest mean is the favorable haplotype under Bed 5IR **(a)**, Bed 2IR **(b)**, and SD **(c)** environments.

The haplotypes and SNPs associated with the *Pi* index were also assessed for their effects on GY *per se* across environments and EYTs (Supplementary Table S8). The favorable haplotype ‘TG’ in block HB11.4 on chromosome 4B showed increased GY from on average 2 to 10% in different environments across EYTs (Supplementary Table S8 and Figure 3). A second favorable haplotype ‘CG’ in block HB14.8 on chromosome 5B increased GY from 0.7 to 9.1%. The favorable alleles in HB1.11 and HB19.8 increased GY from 1.1 to 10.7% and 5 to 11.30%, respectively. The haplotype block HB17.36 showed increased GY across different environments in four EYTs, however, different haplotypes were favorable in different EYTs, indicating G × E. Two more haplotype blocks (HB20.29 on chromosome 7B and HB21.2 on chromosome 7D) exhibited G × E. For the SNPs that were associated in both GWAS analyses for *Pi*, allelic effect ranged from 0.8 to 5.6% (Supplementary Table S8).

### Epistatic Interactions Between Main Effect and Genome-Wide Loci

For GY, epistatic loci interacting with other main effect loci were identified across EYTs for each of the environments (Supplementary Table S9). For example, for GY in Bed-5IR the three haplotype blocks HB4.50, HB5.19 and HB5.21 were interacting in three or four EYT. On average, epistatic interactions explained additional 5 to 9% variation for GY in different environments (Supplementary Table S9). For *Pi*, main epistatic loci were detected on chromosome 4B (HB11.4, HB11.5, HB11.7, HB11.9, and HB11.11) contributing to additional 9% variation (Supplementary Figure S7 and Supplementary Table S10). Higher levels of epistatic interactions were observed between the main effect and genome-wide haplotypes with an average contribution from 9.1 to 16.4% for GY across environments and up to 20% for *Pi* (data not shown). Supplementary Figures S8, S9 show the interactions observed for GY in B-5IR across EYTs and for *Pi*, respectively. Epistatic interactions were also significant among significantly associated SNPs and genome-wide distributed SNPs for GY and *Pi* (data not shown).

### Performance of GS Models

We compared the predictive ability of the four genomic prediction models by incorporating different relationship matrices: Model (1) Genome-wide distributed SNPs (SM model/base model), Model (2) Genome-wide distributed haplotype blocks and epistasis among haplotype blocks (H + E model), Model (3) Genome-wide distributed SNPs, epistasis among SNPs, and single SNPs as fixed effects identified from GWAS and epistasis analyses (SM + E + fixed effects model), Model (4) Genome-wide haplotype blocks, epistasis among haplotypes blocks and haplotypes as fixed effects identified from GWAS and epistasis analyses (H + E + fixed effects model).

**FIGURE 4:**
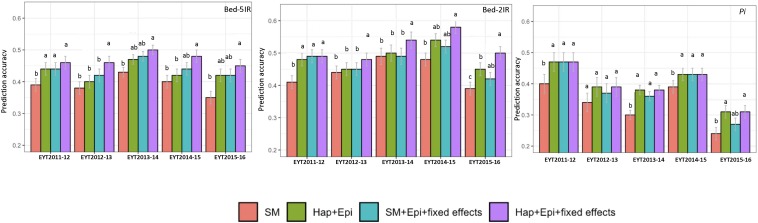
Genomic predictions values for GY (from two illustrative environments B-5IR and B-2IR) and *Pi*. SM, Genome-wide SNPs; H + E, Genome-wide haplotypes + epistatsis; SM + E + GWAS, Genome-wide SNPs + epistasis + fixed effects; and H + E + GWAS, Genome wide haplotypes + epistasis + fixed effects. The whisker line above columns represents LSD values.

The four predication models were applied on two test data sets of GY from B-5IR and B-2IR environments and *Pi*. For GY under B-5IR, the base model showed prediction accuracies from 0.35 to 0.43 (Supplementary Table S11). Incorporating haplotype blocks and epistasis among haplotype blocks (Model 2) into the prediction model resulted in a 3–5% increase in prediction accuracies over the base model (Figure 4). Additionally, accounting for the single marker-based GWAS loci as fixed effects (Model 3) resulted in a similar increase of 5–6% over the base model. The fourth model accounting for the haplotype-based GWAS loci as fixed effects (12 haplotypes used as fixed effects; Table 2) showed increase in accuracies from 7% (EYT 2012-13) to 9% (EYT 2013-14) (Supplementary Table S11 and Figure 4).

**TABLE 2:** Haplotype blocks used as fixed effects in GS models for GY in B-5IR and B2-IR environments. The favorable haplotype(s) is underscored.

	Bed-5IR				Bed-2IR			
			
Haplotype blocks	Haplotypes	Favorable allele effect on mean GY (kg/ha)			Haplotype blocks	Haplotypes	Favorable allele effect on mean GY (kg/ha)	
HB1.10 (1A) S1A_535441795, S1A_535441797	AC GA	EYT2011-12 8,664 8,544	EYT2014-15 5,600 5,446		HB2.23 (1B) S1B_678416771, S1B_678416817	AG GA	EYT2012-13 4,834 4,663	EYT2013-14 3,707 3,636
HB2.5 (1B) S1B_20269363, S1B_20287464	AC GA	EYT2011-12 8,581 8,793	EYT2014-15 5,593 5,678		HB4.30 (2A) S2A_211705334, S2A_211705336	CC TT	EYT2014-15 4,439 4,586	EYT2015-16 3,163 3,228
HB4.38 (2A) S2A_748528721, S2A_748528738	AA GA GG	EYT2012-13 7,744 7,962 7,571	EYT2013-14 6,189 6,149 5,962	EYT2014-15 5,594 5,464 5,285	HB5.1 (2B) S2B_8754756, S2B_8754774	GT TC TT	EYT2011-12 3,690 3,873 3,902	EYT2014-15 4,491 4,588 4,737
HB4.50 (2A) S2A_779846680, S2A_779846718	AA GG	EYT2012-13 7,587 7,781	EYT2013-14 6,056 6,296		HB5.53 (2B) S2B_795302376, S2B_795302390	AT CC	EYT2013-14 3,662 3,774	EYT2014-15 4,582 4,585
HB5.11 (2B) S2B_71692883, S2B_71692929	CC GT	EYT2013-14 6,148 5,964	EYT2014-15 5,485 5,716		HB7.19 (3A) S3A_651647755, S3A_651647756	CG TC	EYT2013-14 3,684 3,774	EYT2014-15 4,509 4,659
HB5.19 (2B) S2B_142247929 S2B_142247930	CA TG	EYT2011-12 8,617 8,476	EYT2014-15 5,645 5,369		HB8.2 (3B) S3B_7031744, S3B_7031759	CC TT	EYT2013-14 3,770 3,624	EYT2014-15 4,560 4,411
HB5.21 (2B) S2B_178761042, S2B_178771120	CC CT GC	EYT2012-13 7,750 7,780 7,634	EYT2013-14 6,000 6,226 6,086		HB8.28 (3B) S3B_755079571, S3B_755079586	CA TT	EYT2011-12 3,726 3,585	EYT2014-15 4,649 4,513
HB11.3 (4B) S4B_4128125, S4B_4128135	CG GC	EYT2012-13 7,799 7,677	EYT2015-16 6,949 7,153		HB14.16 (5B) S5B_387952822, S5B_387952832	CA TT	EYT2013-14 3,646 3,754	EYT2015-16 3,168 3,239
HB14.38 (5B) S5B_571861468, S5B_571861476	GA TG	EYT2011-12 8,476 8,768	EYT2013-14 6,016 6,180		HB17.1 (6B) S6B_3567046, S6B_3567059, S6B_3567083	CAA CAC TTC	EYT2013-14 3,723	
HB19.8 (7A) S7A_36087756, S7A_36087757, S7A_36096388	AGA AGG GAG	EYT2011-12 8,569 8,191 8,687	EYT2013-14 5,859 5,894 6,166		HB19.39 (7A) S7A_675454642, S7A_675454643	CT TG	EYT2013-14 3,729 3,671	EYT2015-16 3,157 3,266
HB20.12 (7B) S7B_160614818, S7B_160614819	CG TA	EYT2011-12 8,690 8,558	EYT2014-15 5,689 5,554					
HB20.41 (7B) S7B_724005624, S7B_724005636, S7B_724005641	ATA GCG GTG	EYT2013-14 5,989 6,157 6,263	EYT2014-15 5,413 5,615 5,678					

*The markers included in haplotype blocks are shown under block names. The number in marker name represents physical positions of SNPs based on wheat reference genome vs. 1.0.*

For GY in B-2IR, calculated prediction accuracies were overall higher than in B-5IR and ranged from 0.39 to 0.48 (Supplementary Table S11) for the base model. Model 2 resulted in a 2–5% increase in prediction accuracy (Figure 4). Model 3 did not provide any advantage in EYT2013-14, EYT2014-15 and EYT2015-2016 over Model 2. The fourth model in which 10 haplotypes were used as fixed effects (Table 2) proved again to be the best model in increasing the prediction accuracies to up to 10% over the base model in three EYTs (EYT2011-12, EYT2014-15 and EYT2015-16).

For *Pi* although a clear trend was not observed, two scenarios were noteworthy; when across environment heritability of GY was high (>0.55) for instance in EYT2011-12 and EYT2014-15 (Table 1), no variation was observed among models 2, 3, and 4 and they performed equally well over the base model in EYTs (Figure 4 and Supplementary Table S12). However, for EYTs with a lower across environment heritability (<0.55), both models based on haplotypes (Models 2 and 4) performed slightly better than the remaining two models.

## Discussion

While trying to identify the genetic determinants of complex traits such as GY using GWAS approach, common genetic variants (5–95% frequencies) with small phenotypic effects are identified and rare variants (<1% frequencies) of large effects remain unidentified. Furthermore, despite the increasing awareness that epistasis forms the genetic basis of complex traits, the contribution of epistasis in the genetics of GY has been rarely investigated in GWAS studies (Mackay, 2014; Lachowiec et al., 2015). Hence, complete genetic architecture of the trait remains hidden leading to ‘missing heritability’ issues. Of the various suggested approaches to deal with ‘missing heritability’ including large panel sizes as large as 10,000 to identify rare variants and whole genome sequencing to cover both causative variants and LD-linked variants, focusing on haplotypes-based GWAS and estimation of epistatic interactions can provide immediate and inexpensive solutions (Lachowiec et al., 2015). Use of multi-allelic haplotypes has significantly improved the power and robustness of GWAS studies in crops (Qian et al., 2017) including soybean (Hao et al., 2012), barley (Lorenz et al., 2010) maize (Lu et al., 2012), and durum wheat (N’Diaye et al., 2017).

We found 58 haplotypes associated with GY of which 16 associations were ‘environment-specific’ and 26 were more ‘robust’ across environments and/or years. Many of the genomic regions identified in the two different drought stress environments (B-2IR and SD representing mild and severe drought stress, respectively) were different from each other, pointing to the additional complexity of drought stress tolerance. On chromosome 6B, a major GY QTL (HB17.1) was identified with the favorable haplotype ‘TAC’ showing an allele effect of up to +530 and +303 kg/ha under B-2IR and SD, respectively. A recent study reported two meta-QTL for adaptation to drought stress on chromosome 6B in wheat (Acuna-Galindo et al., 2015). The QTL detected here is ∼15 cM away from these meta-QTL and therefore likely novel. For HS, ‘environment specific’ GY QTL were identified on chromosomes 2B (HB5.42), 3B (HB8.26) and 7B (HB20.38) of which HB8.26 showed the largest effect on GY. The favorable allele ‘GT’ led to increased GY of 189 – 374 kg/ha across EYTs. On chromosome 3B, four meta-QTL for adaptation to combined heat and drought stresses were mapped previously (Acuna-Galindo et al., 2015). The QTL identified in this study is very close to one of these metaQTL (within 5 cM). In durum wheat, QTL hotspots for GY and GY components in high yield potential, drought, and heat stress environments were reported on chromosomes 2A and 2B (Sukumaran et al., 2018a, b).

In a previous study, we identified genomic regions associated with yield stability using the superiority index *Pi* in one of the EYTs studied here and assessed the effects of *Pi-*associated loci on GY in multiple environments (Sehgal et al., 2017). In this study, which is first of its own kind, two different stability indices (*Pi* and ER) were compared on such a large dataset. Most and almost all studies in crops have done such comparisons in a handful of cultivars or varieties (Scapim et al., 2000; Temesgen et al., 2015). The ability of *Pi* to select both high yielding and stable genotypes (Supplementary Figure S3) re-established that *Pi* is a suitable parameter for selecting widely adapted high yielding wheat genotypes. Fifteen haplotype blocks were identified to be associated with *Pi* on chromosomes 1A, 4A, 4B, 5B, 6B, 7A, 7B, and 7D. Of these, eight haplotype blocks were on chromosomes 4B and 7B, indicating ‘hot spot’ regions for yield stability. Three haplotype blocks on chromosome 7B (HB20.29, HB20.33, and HB20.38) coincided with QTL already reported in various studies and environments (Quarrie et al., 2005; Paliwal et al., 2012; Sehgal et al., 2017), while HB20.6 and HB20.14 are novel with favorable effects on GY in three (up to 8.9%) and six (up to 11.3%) trials, respectively. On chromosome 4B, a major genomic region was identified with three haplotype blocks (HB11.4, HB11.5, and HB11.7; altogether 480 Mb long) associated with *Pi*, in which haplotype ‘TG’ increased GY in nine trials. On chromosome 4B, two meta-QTL have been reported on its long arm (Zhang et al., 2010). The haplotype blocks identified here mapped on chromosome 4BS. It could be speculated that the QTL could be a pleiotropic effect of the *Rht-B1* gene also located on chromosome 4BS. However, the dwarfing allele *Rht-B1a* is usually fixed (95–97% across the five EYTs) in CIMMYT wheat germplasm. The QTL on 4B and 7B provide new genomic regions for subsequent analyses of their underlying candidate genes.

We compared the results obtained by haplotype-based GWAS with single marker-based GWAS, which has rarely been investigated in wheat (N’Diaye et al., 2017). The haplotype-based analysis resulted in an increase of the phenotypic variance explained for both GY and *Pi* when compared to single marker analysis. These results are similar to previous studies in other crops and durum wheat (Lorenz et al., 2010; Hao et al., 2012; Lu et al., 2012; N’Diaye et al., 2017). We identified a lower number of haplotypes in comparison to single SNPs associated with the traits, but more haplotypes with favorable effects across EYTs and environments. For example for GY, haplotype blocks HB20.41 in B-5IR, HB5.1 and HB8.28 in B-2IR, and for *Pi* haplotype blocks HB11.4, HB11.7, and HB21.2.

Epistasis plays a significant role in the genetic architecture of complex traits, but its contribution has not been investigated in depth using GWAS approaches. In soft winter wheat, Reif et al. (2011) investigated the role of epistasis in a GWAS study for GY and reported that main effects dominated the genetic architecture of GY and epistatic interactions contributed only little. In contrast, in our studies (Sehgal et al., 2016, 2017), GY and *Pi* were controlled by both main and epistatic effects. Reif et al. (2011) used a smaller and a narrower panel of elite breeding lines (455 lines derivatives from a few parents) adapted to central European conditions. A smaller number of alleles fixed in the germplasm panel might have reduced epistasis among loci. In this study, we investigated a significant larger set of breeding lines selected from a wide range of genetic backgrounds and for a number of diverse mega-environments globally.

Since we observed several SNPs and haplotypes associated to GY and *Pi* significantly associated across some environments and EYTs, we tested whether these QTLs could affect prediction accuracy of these complex traits. We tested this approach for GY in two illustrative environments (B-5IR and B-2IR) and for *Pi*. For GY, a general trend observed was that the model accommodating haplotype-based GWAS loci and epistatic effects was superior to other models tested and resulted in up to 9 and 10% increase in prediction accuracies in B-5IR and B-2IR environments, respectively. These results indicate that if genomic regions with moderate effects but showing significant associations across years and/or environments are identified for a complex trait, using them as fixed effects can lead to better performance of GS models. Up to date, several different strategies were tested at CIMMYT to increase prediction accuracies for GY, for example, using pedigrees and markers individually and combined, G × E models and incorporating additional secondary traits (Crossa et al., 2007, 2010, 2011; Burgueño et al., 2012; Rutkoski et al., 2016). This is the first report to incorporate the genetic architecture into GS models. The increase in prediction accuracies for GY is similar to that observed in previous studies in wheat mentioned above. Thus, there is potential for combining the various approaches and further explore their effect on prediction accuracies.

Including the epistatic effects and single marker-based GWAS results as fixed effects in the GS prediction models also increased prediction accuracies in comparison to the base model but not to the same extent as haplotypes. Two recent studies in rice (Spindel et al., 2016) and maize (Bian and Holland, 2017) reported increases in prediction accuracies using SNPs as fixed effects. The fact that we found less robust SNPs, with additional minor effects on GY and *Pi* are the most likely reason for this finding (Boichard et al., 2012).

Prediction accuracies for *Pi* index were in a similar range to those for GY. However, when GS models were tested for the *Pi* index, the increase in prediction accuracy by incorporating GWAS results could not be repeated. The genetic architecture of yield stability is difficult to capture and we speculate that this higher complexity of the trait has led to our results. This is for example highlighted by the finding of several haplotype blocks were associated with *Pi*, but failed to show a consistent favorable allele. Furthermore, simulation studies have shown that the number of independent chromosome segments that enters into GS models influences the estimate of accuracy in fixed effects models (Brard and Richard, 2015). The main effect haplotypes for *Pi* were from only three chromosomes (4A, 4B, and 7B) and the epistatically interacting loci were from chromosome 4B (HB11.5, HB11.7, HB11.9, HB11.11). The lack of sufficient haplotype blocks across the genome found in this study might be the reason for the observed results.

We conclude that the utility of GS incorporating GWAS results is noteworthy for GY when GWAS results identify highly significant and robust genomic regions. GS predictions were even higher when haplotypes instead of SNP were used as fixed effects. With the upsurge in dense marker data sets coming from different genotyping platforms leading to more markers than observations (Winfield et al., 2016), haplotypes-based dissection of genetic architecture seems more useful and practical for both reliable gene discovery and genomic predictions. Although further research is needed, our results suggest incorporating this approach in GS deployment.

## Data Availability Statement

The datasets analyzed for this study can be found in the CIMMYT Dataverse (http://hdl.handle.net/11529/10548366).

## Author Contributions

DS and SD conceived the manuscript. SD designed the research. DS and UR analyzed the data. DS wrote the manuscript. SM and RS generated phenotypic data. JP generated allele called GBS marker data. All authors reviewed the manuscript.

## Conflict of Interest

The authors declare that the research was conducted in the absence of any commercial or financial relationships that could be construed as a potential conflict of interest.
