# Supplementary material for: Incorporating Genome-Wide Association Mapping Results Into Genomic Prediction Models for Grain Yield and Yield Stability in CIMMYT Spring Bread Wheat
Source: Front Plant Sci. 2020 Mar 4;11:197. doi: 10.3389/fpls.2020.00197 (PMC7064468; doi:10.3389/fpls.2020.00197)
Supplement: Supplementary file 1 [file Data_Sheet_1.zip › Figures S1-9.PDF]

Incorporating Genome-wide Association Mapping Results into Genomic Prediction Models for Grain Yield and Yield Stability in CIMMYT Spring Bread Wheat

Deepmala Sehgal[1], Umesh Rosyara[1], Suchismita Mondal[1], Ravi Singh[1], Jesse Poland[2], Susanne Dreisigacker[1, *]

[1]International Center for Maize and Wheat Improvement (CIMMYT), Km. 45, Carretera Méx-Veracruz, El Batán, Texcoco, México, CP 56237

[2]Kansas State University, Manhattan, Kansas 66506, United States

Corresponding author

Susanne Dreisigacker
Wheat Molecular Breeding Global Wheat Program
International Center for Maize and Wheat Improvement (CIMMYT)
Km. 45, Carretera Méx-Veracruz,
El Batán, Texcoco, México, CP 56237
Email: s.dreisigacker@cgiar.org
Telephone: +52 55 5804 2004 ext. 2184

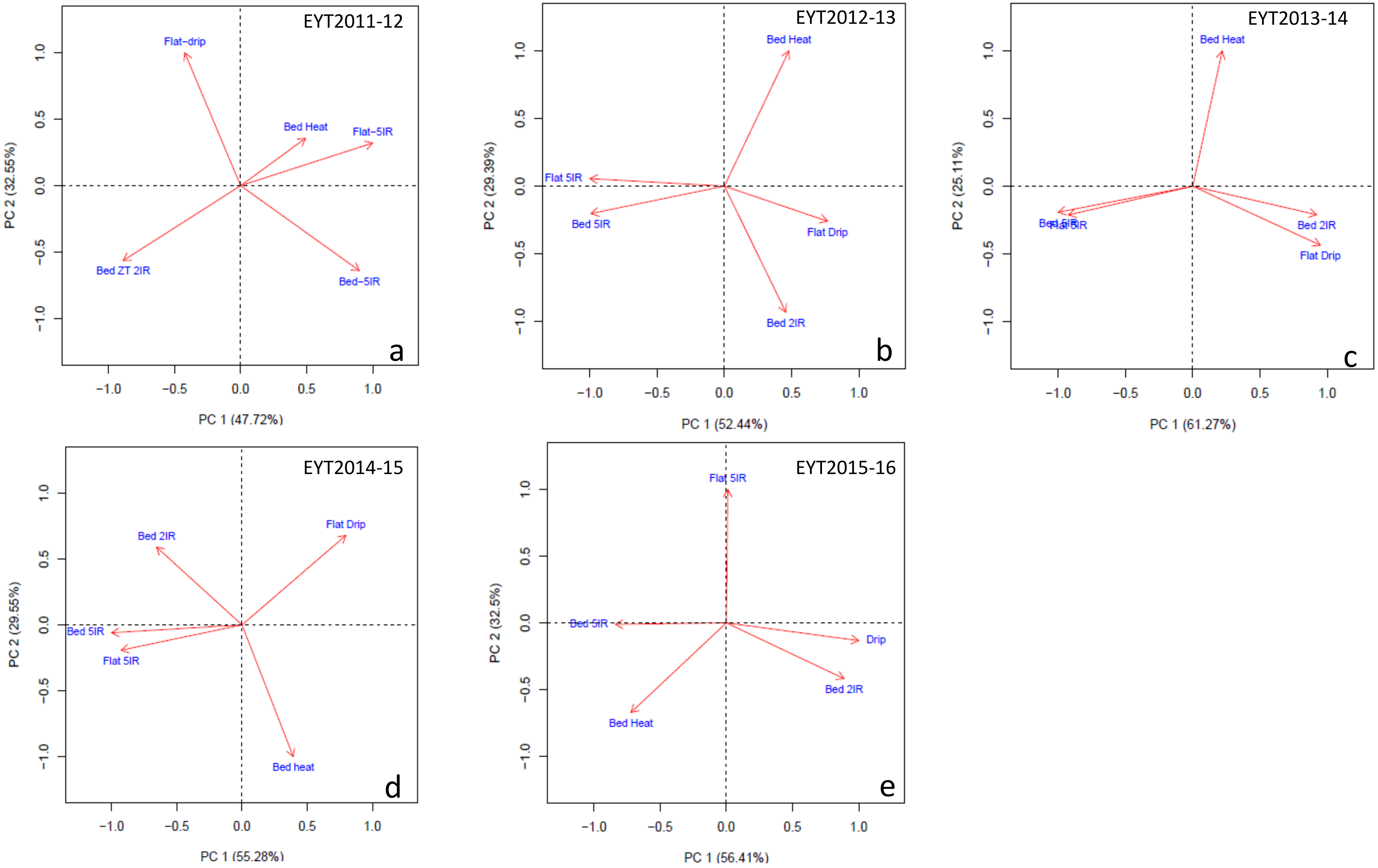

Fig. S1 GGE biplots of five contrasting environments in all EYTs; EYT2011-12 (a), EYT2012-13 (b), EYT2013-14 (c), EYT2014-15 (d) and EYT2015-16 (e)

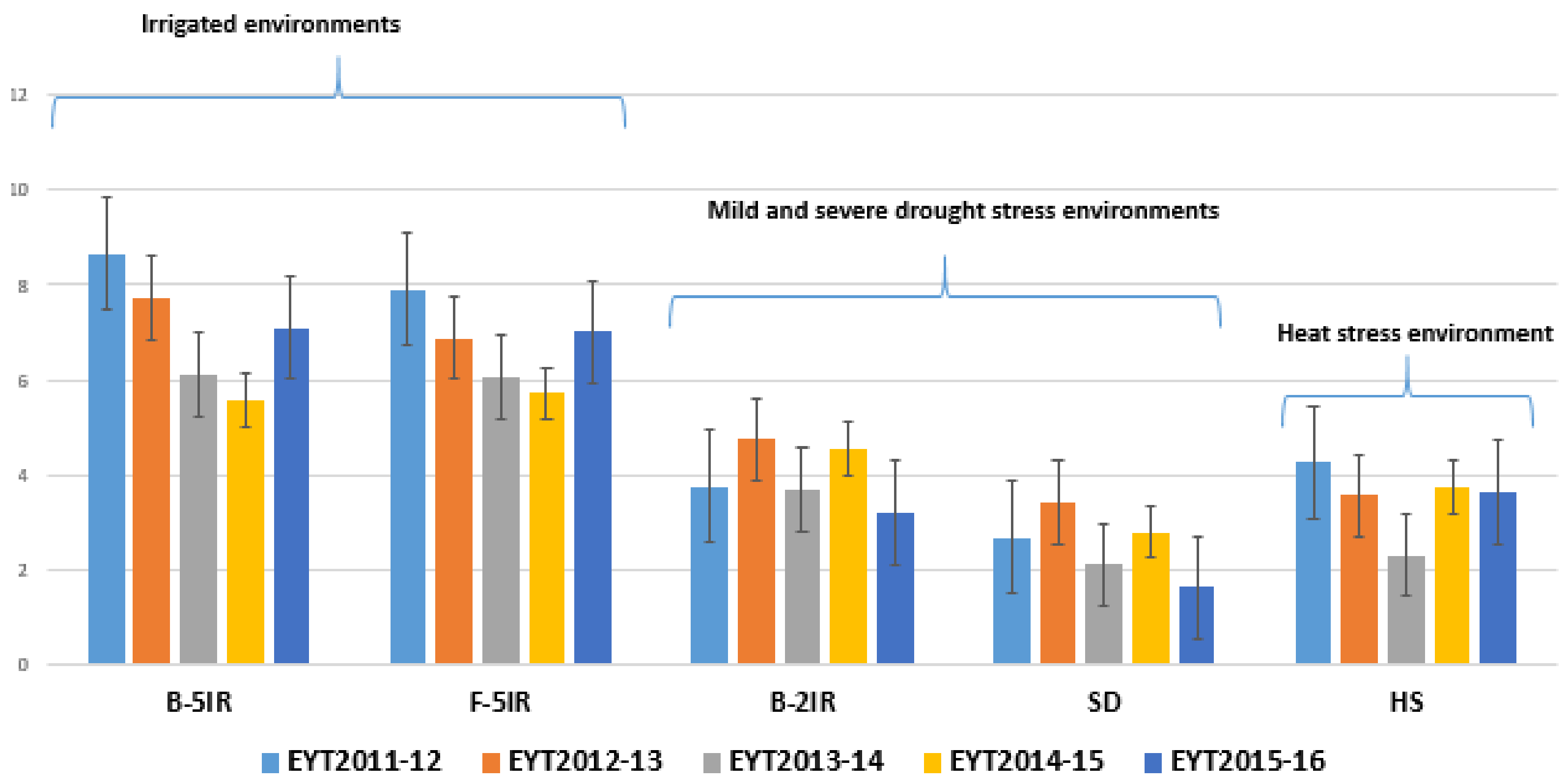

Fig. S2 Adjusted mean of GY in each of the five environments in five EYTs

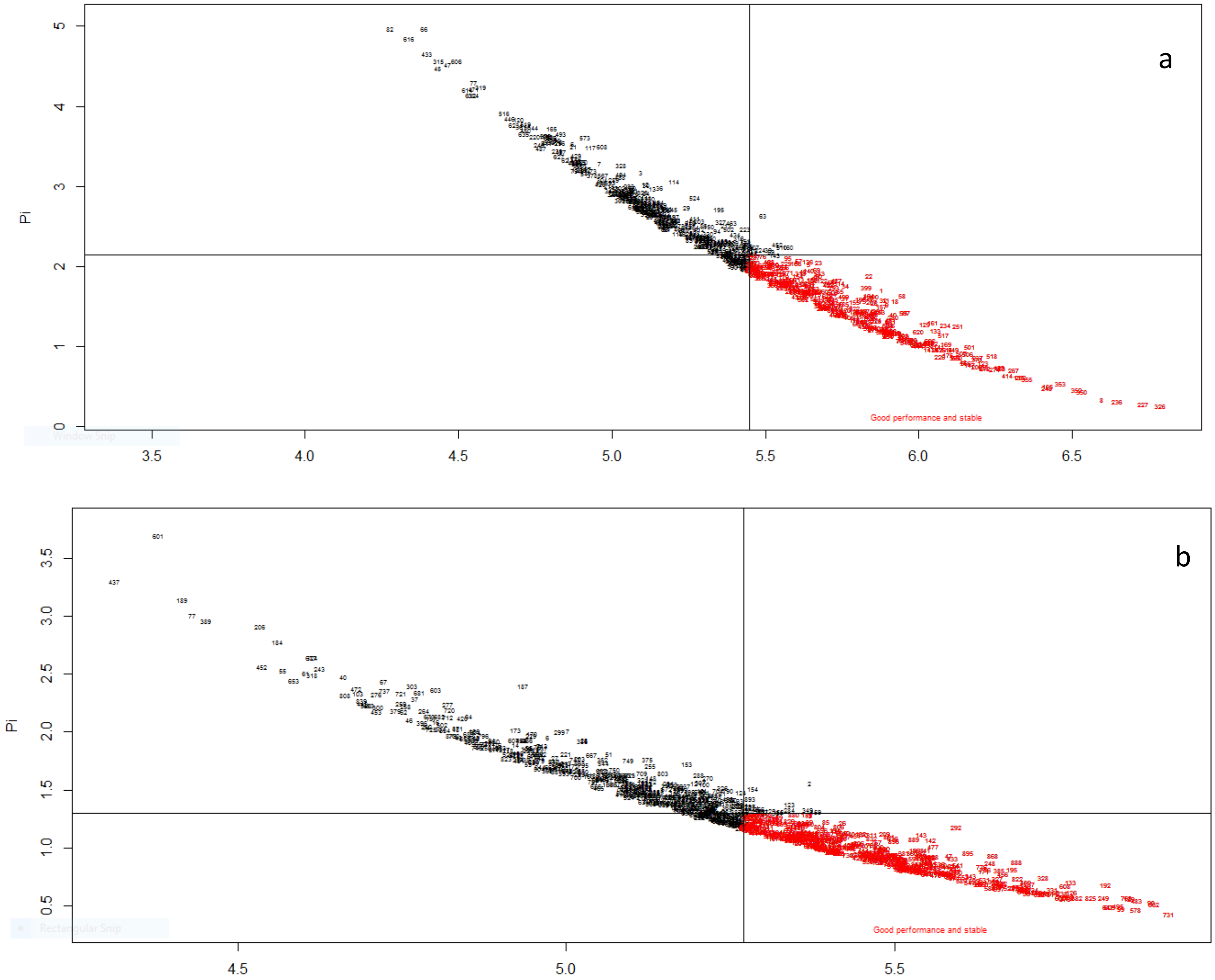
a
Pi
0
1
2
3
4
5
3.5
4.0
4.5
5.0
5.5
6.0
6.5
Good performance and stable
b
Pi
0.5
1.0
1.5
2.0
2.5
3.0
3.5
4.5
5.0
5.5
Good performance and stable

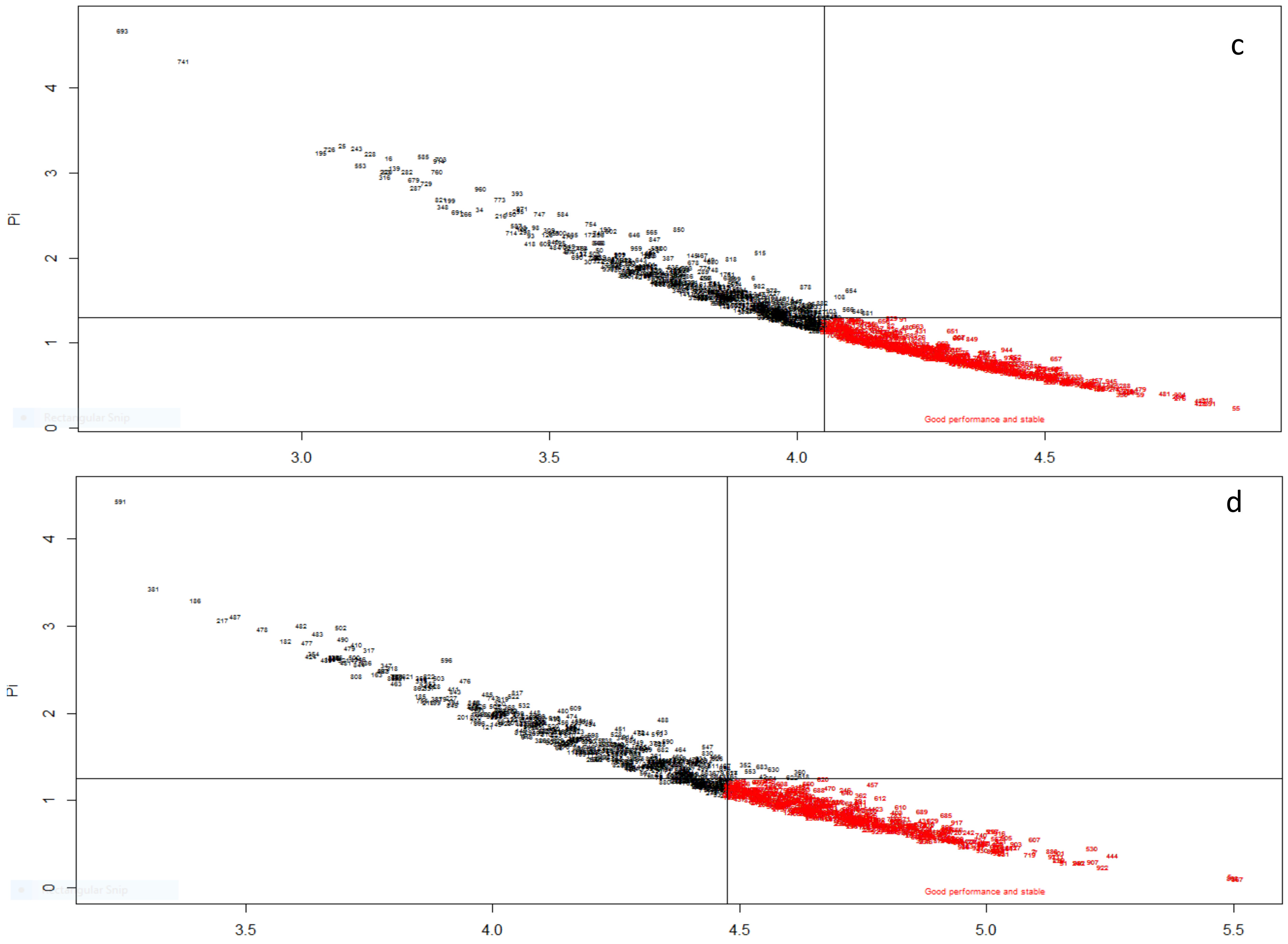
c
Pi
0
1
2
3
4
3.0
3.5
4.0
4.5
Good performance and stable
d
Pi
0
1
2
3
4
3.5
4.0
4.5
5.0
5.5
Good performance and stable

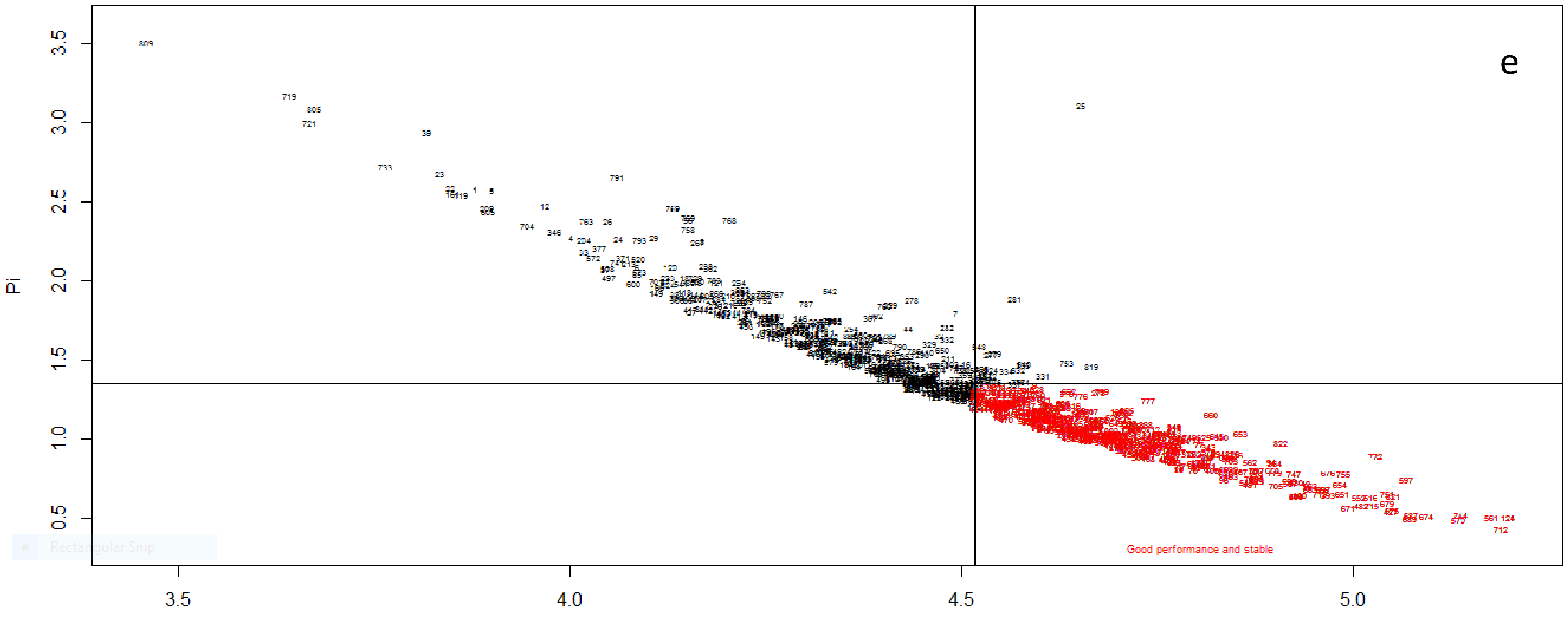

Fig. S3 G x E plots for *Pi* vs mean GY across environments for EYT2011-12 (a), EYT2012-13 (b), EYT2013-14 (c), EYT2014-15 (d) and EYT2015-16 (e). The lower right side of the plot shows good performing and stable lines in red.

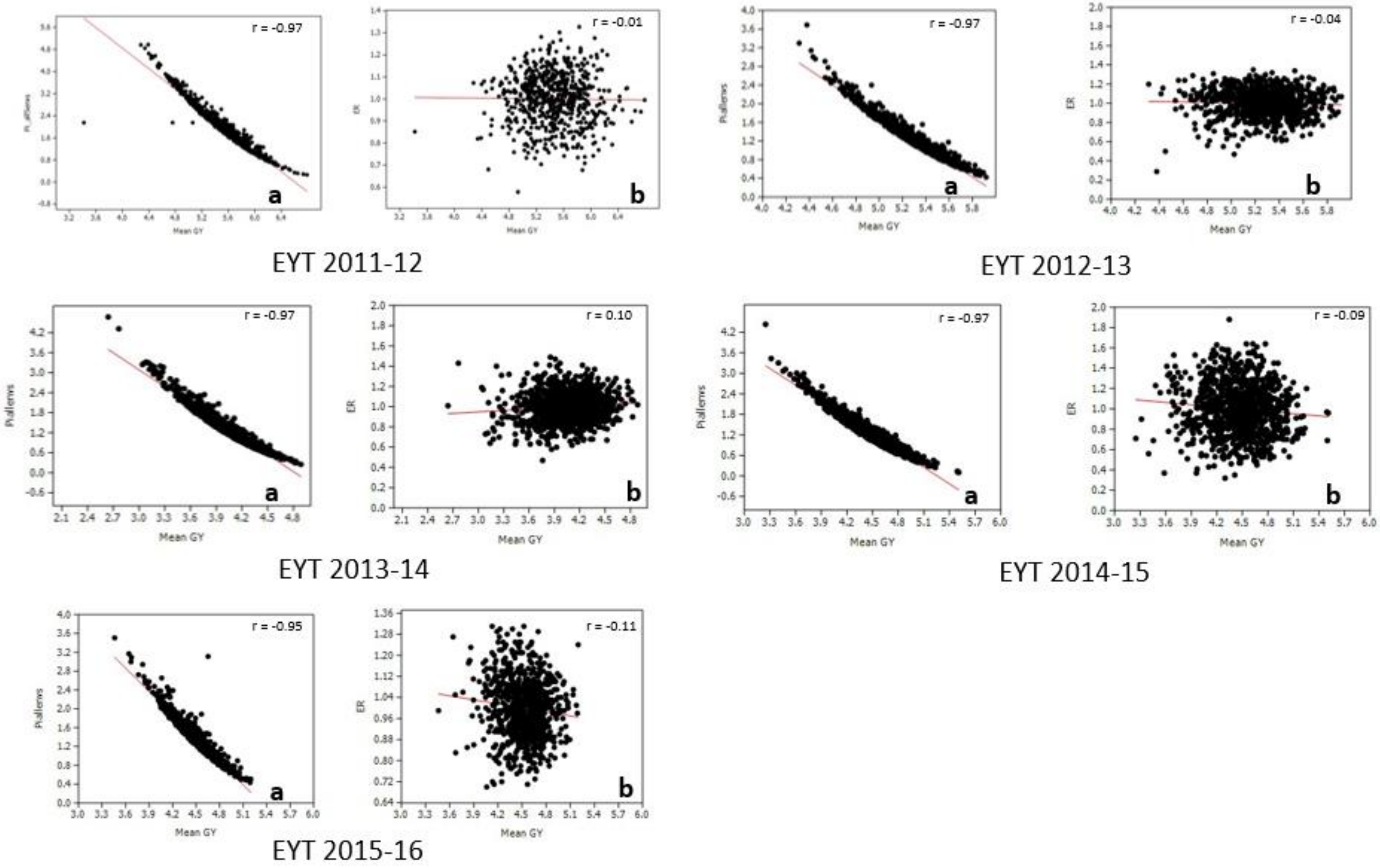

Fig. S4 Genetic correlation between mean GY and Pi (a) and between mean GY and ER (b) in all EYTs.

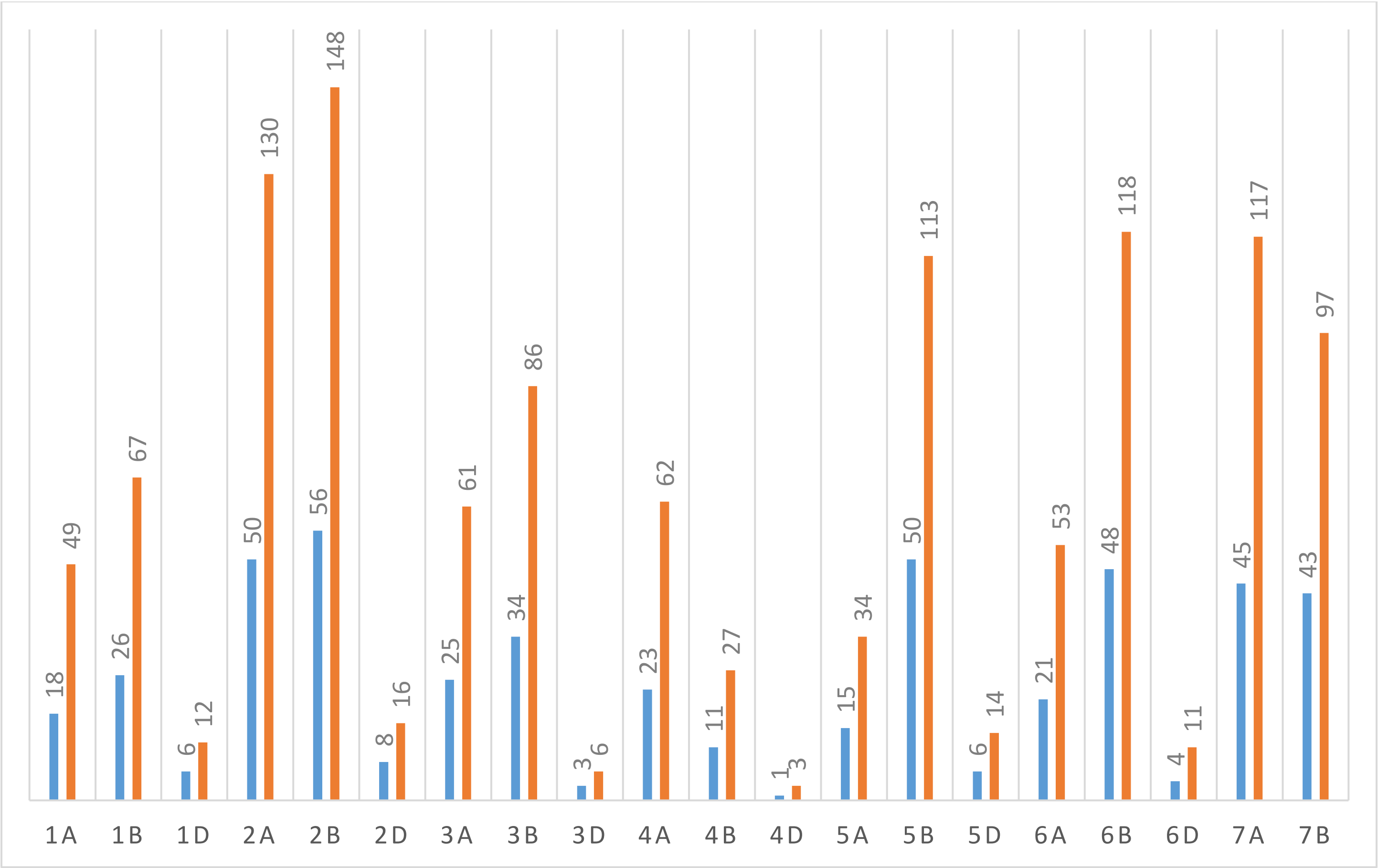

Fig. S5 Total number of haplotype blocks (blue) and haplotypes (orange) chromosome wise

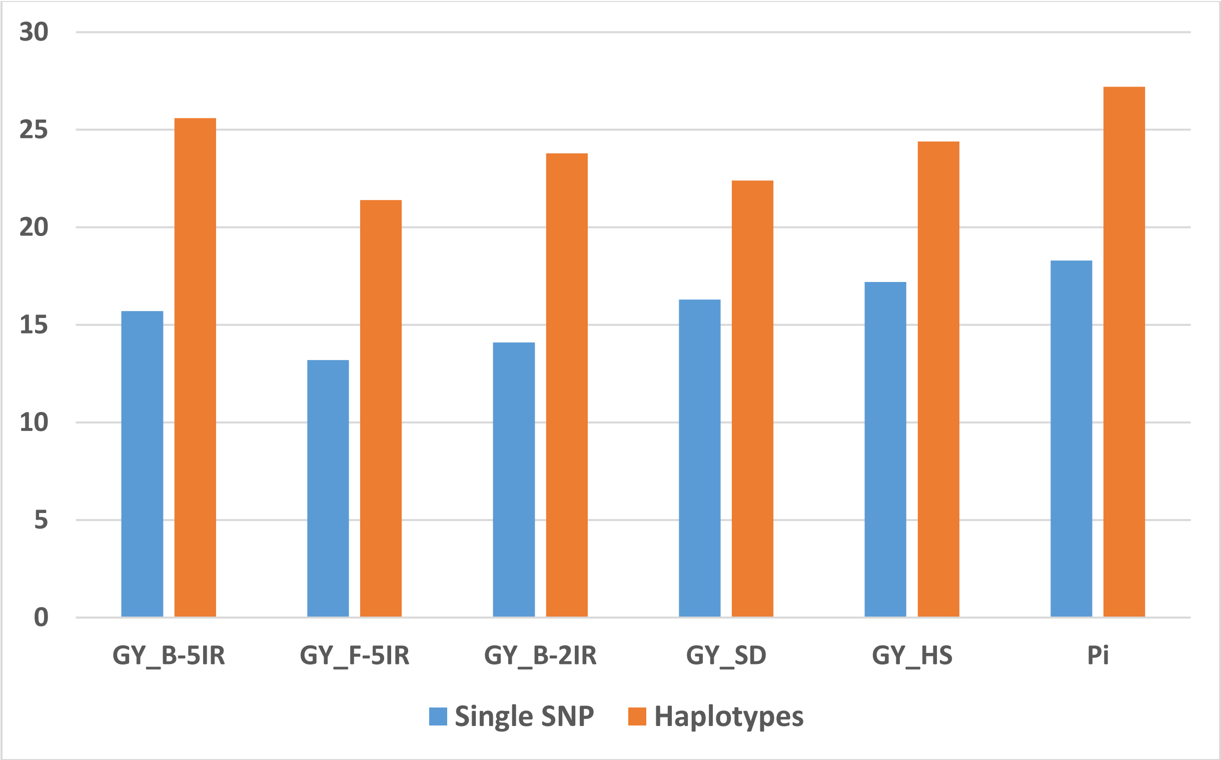

Fig. S6 Percentage variation (R^2) explained by associated SNPs (blue) and haplotype blocks (orange)

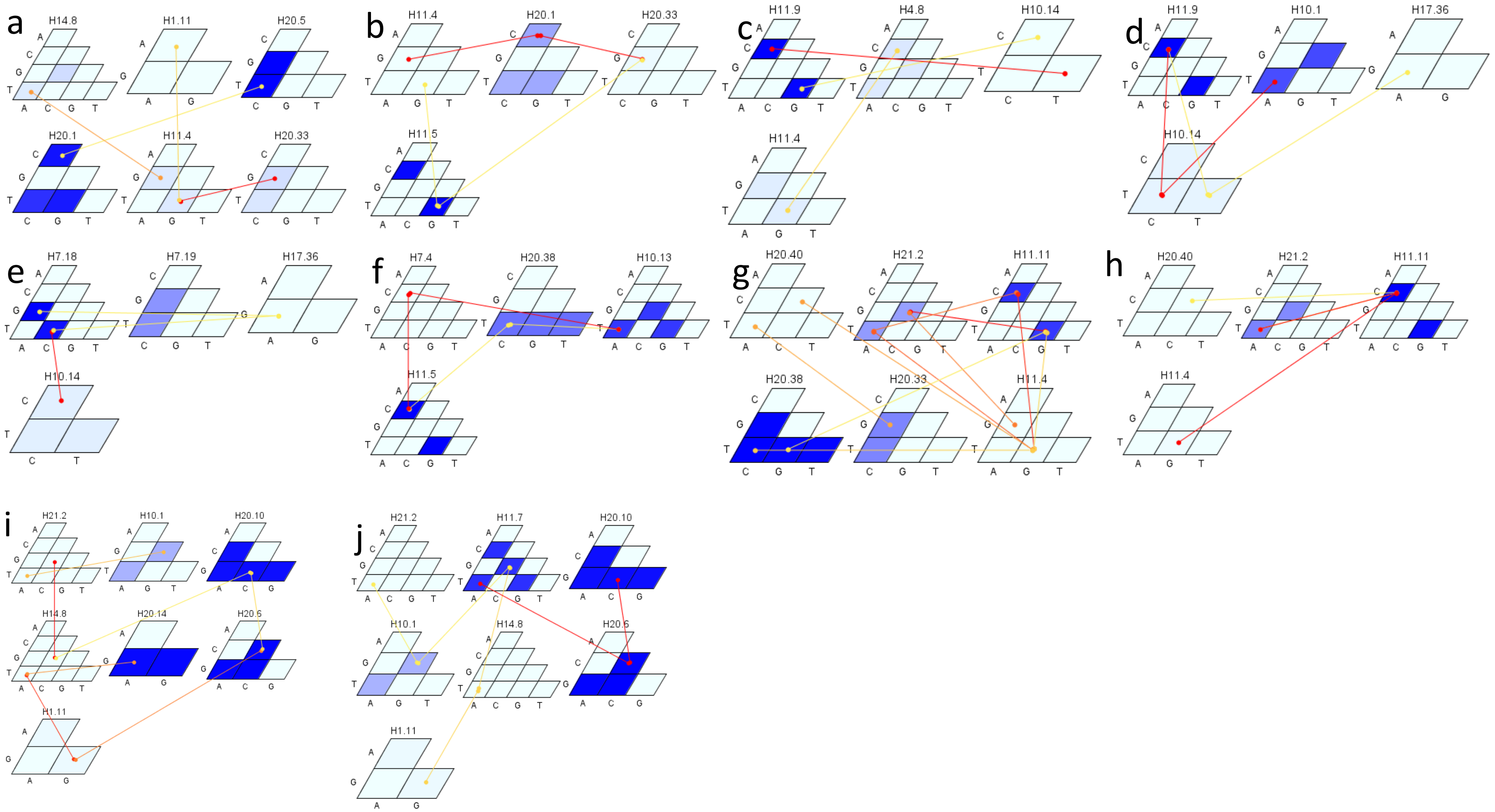

Fig. S7 Epistatic interactions among haplotypes with main effects for *Pi* in EYT2011-12 (a: two locus b: three locus), EYT2012-13 (c: two locus d: three locus), EYT2013-14 (e: two locus f: three locus), EYT2014-15 (g: two locus h: three locus) and EYT2015-16 (i; two locus j: three locus).

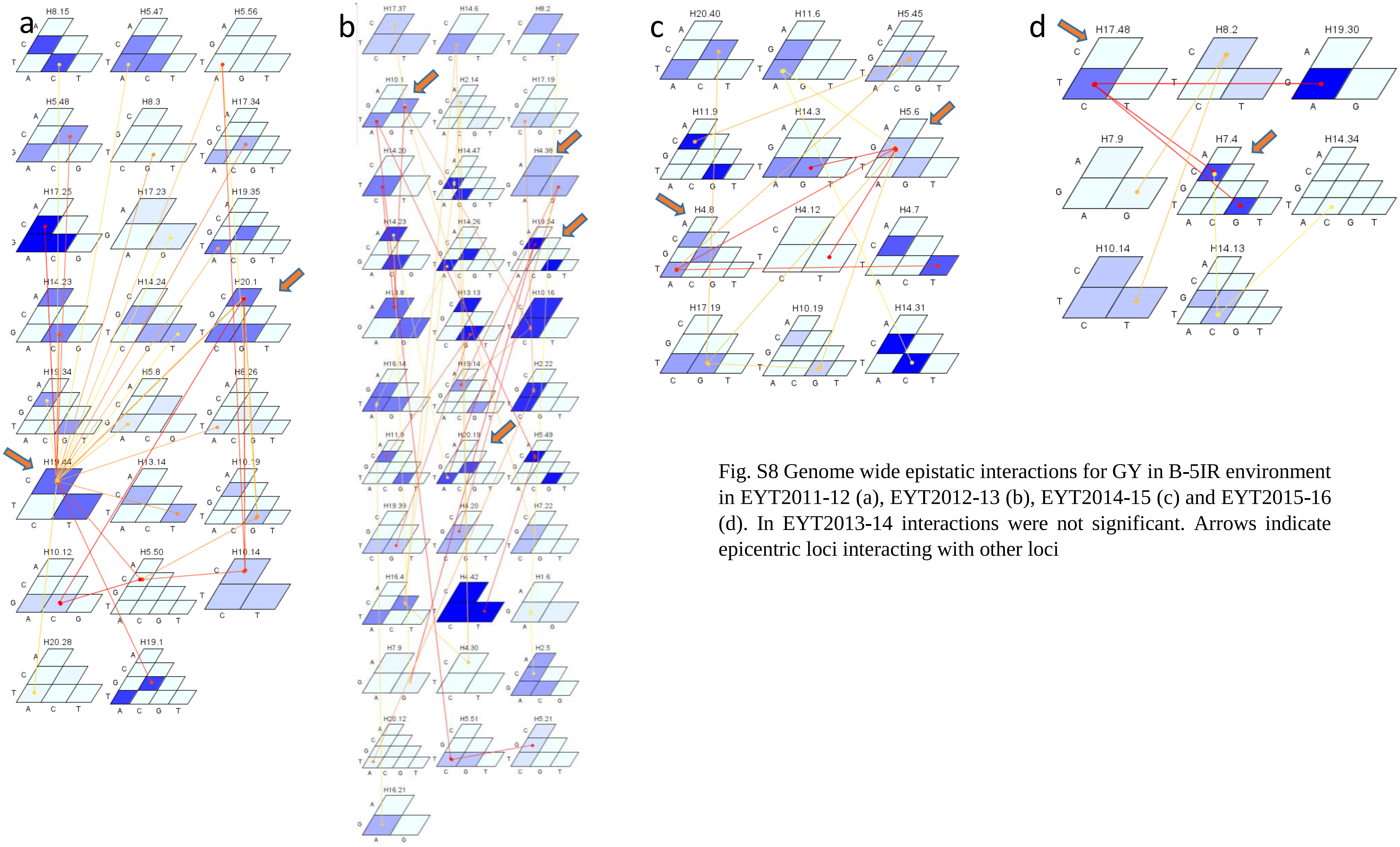

Fig. S8 Genome wide epistatic interactions for GY in B-5IR environment in EYT2011-12 (a), EYT2012-13 (b), EYT2014-15 (c) and EYT2015-16 (d). In EYT2013-14 interactions were not significant. Arrows indicate epicentric loci interacting with other loci

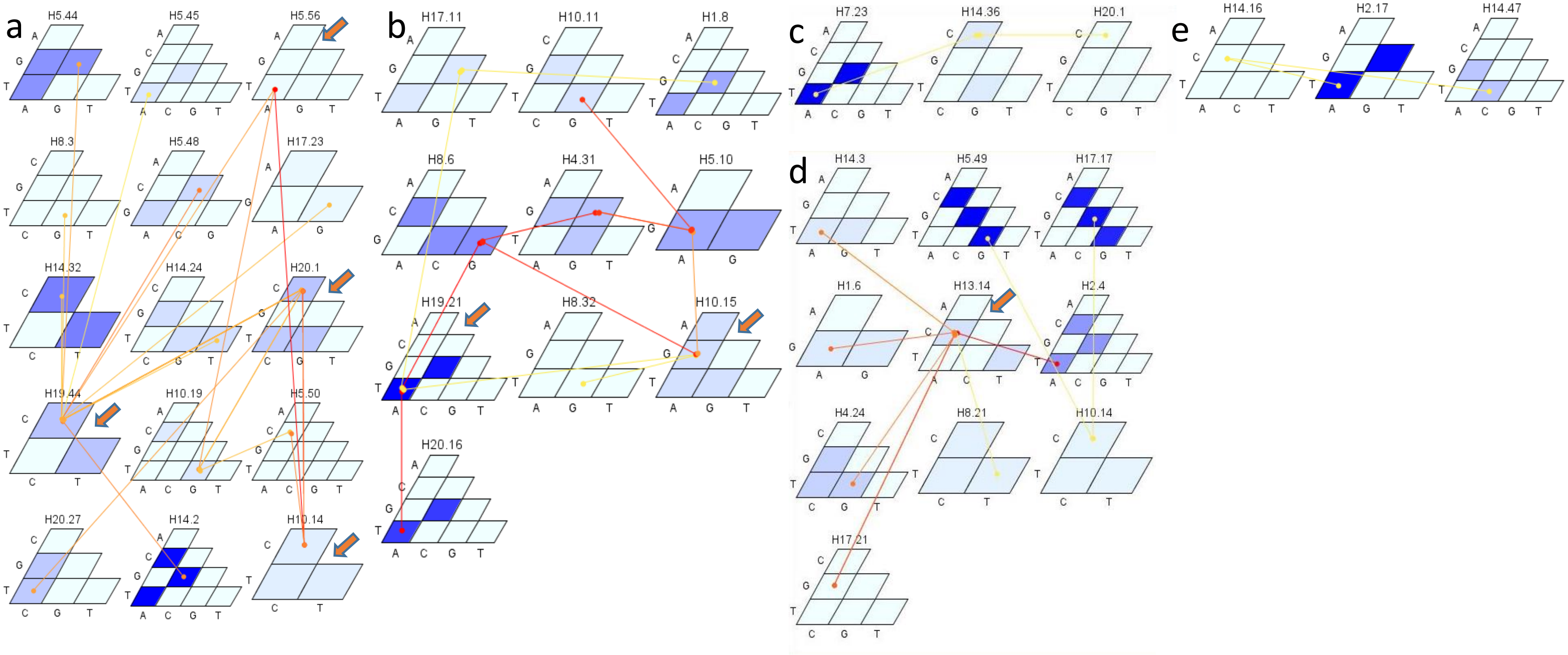

Fig. S9 Genome wide epistatic interactions for *Pi* in EYT2011-12 (a), EYT2012-13 (b), EYT2013-14 (c), EYT2014-15 (d) and EYT2015-16 (e). Arrows indicate epicentric loci
