# Supplementary material for: Incorporating Genome-Wide Association Mapping Results Into Genomic Prediction Models for Grain Yield and Yield Stability in CIMMYT Spring Bread Wheat
Source: Front Plant Sci. 2020 Mar 4;11:197. doi: 10.3389/fpls.2020.00197 (PMC7064468; doi:10.3389/fpls.2020.00197)
Supplement: Supplementary file 1 [file Data_Sheet_1.zip › Table S10.pdf]

S10 Table Two and three locus epistatic interactions among main effect haplotypes for *Pi* in five EYTs. The underlined haplotypes shows epicentric loci.

Pi	Interacting alleles	R^2 (%)
EYT2011-12	H20.33(CG) <u>H11.4(GT)</u>	6.3
	H14.8(AT) <u>H11.4(AG)</u>	5.6
	H20.5(CT) H20.1(CC)	7.9
	<u>H11.4(GT)</u> H1.11(AA)	5.4
	H20.33(CG) H20.1(CC) <u>H11.4(AG)</u>	9.9
	H20.33(CG) H11.5(GT) <u>H11.4(GT)</u>	8.8
EYT2012-13	<u>H11.4(GT)</u> H4.8(AC)	0.4
	H10.14(CC) H11.9(GT)	0.2
	H10.14(TT) H11.9(AC)	0.5
	H17.36(AG) H10.14(TT) H11.9(AC)	0.7
	H10.1(AT) H10.14(CT) H11.9(AC)	0.5
EYT2013-14	H10.14(CC) H7.18(CT)	4.0
	H17.36(AG) H7.18(CT)	2.8
	H17.36(AG) H7.18(AG)	2.8
	H11.5(AC) H20.38(CT) H10.13(AT)	2.9
	H11.5(AC) H7.4(AC) H10.13(AT)	3.1
EYT2014-15	H21.2(CG) H11.11(GT)	4.1
	H21.2(CG) <u>H11.4(AG)</u>	2.3
	H21.2(AT) H11.11(AC)	2.7
	H21.2(AT) <u>H11.4(GT)</u>	2.6
	H20.33(CG) H20.40(AT)	6.0
	H11.11(AC) <u>H11.4(GT)</u>	2.7
	H11.11(GT) H20.38(GT)	1.7
	H21.2(AT) H11.11(AC) H20.40(CC)	7.4
	H21.2(AT) H11.11(AC) <u>H11.4(GT)</u>	5.9
EYT2015-16	H1.11(GG) H14.8(AT)	2.4
	H1.11(GG) H20.6(CC)	3.4
	H14.8(CG) H21.2(CG)	1.8
	H14.8(CG) H20.10(CG)	2.7
	H14.8(AT) H20.14(AG)	2.6
	H21.2(AT) H10.1(GG)	2.5
	H20.6(CC) H20.10(CG)	3.8
	H1.11(GG) H14.8(AT) H11.7(CG)	5.3
	H21.2(AT) H10.1(GG) H11.7(CG)	6.4
	H11.7(AT) H20.6(CC) H20.10(CG)	15.8
