# Supplementary material for: Incorporating Genome-Wide Association Mapping Results Into Genomic Prediction Models for Grain Yield and Yield Stability in CIMMYT Spring Bread Wheat
Source: Front Plant Sci. 2020 Mar 4;11:197. doi: 10.3389/fpls.2020.00197 (PMC7064468; doi:10.3389/fpls.2020.00197)
Supplement: Supplementary file 1 [file Data_Sheet_1.zip › Table S11.pdf]

S11 Table Prediction accuracies of four GS models for GY in two specific environments

GY_B-5IR	SM	H+E	SM + E + fixed effects	H+E+fixed effects	GY_B-2IR	SM	H+E	SM + E + fixed effects	H+E+fixed effects
EYT2011-12	0.39(0.07)	0.44(0.06)	0.44(0.05)	0.46(0.06)	EYT2011-12	0.41(0.08)	0.46(0.04)	0.48(0.08)	0.51(0.08)
EYT2012-13	0.38(0.07)	0.40(0.08)	0.42(0.05)	0.43(0.09)	EYT2012-13	0.44(0.06)	0.44(0.09)	0.46(0.08)	0.48(0.08)
EYT2013-14	0.43(0.05)	0.46(0.07)	0.48(0.06)	0.51(0.06)	EYT2013-14	0.48(0.06)	0.50(0.07)	0.49(0.08)	0.53(0.05)
EYT2014-15	0.40(0.07)	0.42(0.06)	0.46(0.05)	0.48(0.09)	EYT2014-15	0.48(0.04)	0.54(0.06)	0.53(0.06)	0.58(0.08)
EYT2015-16	0.35(0.09)	0.42(0.06)	0.42(0.05)	0.44(0.06)	EYT2015-16	0.39(0.07)	0.45(0.05)	0.44(0.08)	0.50(0.09)
All	0.28(0.06)	0.31(0.07)	0.31(0.06)	0.33(0.08)	All	0.29(0.08)	0.34(0.06)	0.35(0.06)	0.36(0.07)
