# Supplementary material for: Incorporating Genome-Wide Association Mapping Results Into Genomic Prediction Models for Grain Yield and Yield Stability in CIMMYT Spring Bread Wheat
Source: Front Plant Sci. 2020 Mar 4;11:197. doi: 10.3389/fpls.2020.00197 (PMC7064468; doi:10.3389/fpls.2020.00197)
Supplement: Supplementary file 1 [file Data_Sheet_1.zip › Table S12.pdf]

S12 Table Prediction accuracies of four GS models for *Pi*

Pi	SM	H+E	SM + E + fixed effects	H+E+fixed effects
EYT2011-12	0.4(0.04)	**0.47(0.05)**	**0.47(0.05)**	**0.47(0.04)**
EYT2012-13	0.34(0.04)	0.39(0.04)	0.37(0.03)	0.39(0.03)
EYT2013-14	0.3(0.04)	0.38(0.05)	0.36(0.05)	0.38(0.05)
EYT2014-15	0.39(0.03)	**0.43(0.02)**	**0.43(0.03)**	**0.43(0.02)**
EYT2015-16	0.24(0.03)	0.31(0.05)	0.27(0.06)	0.31(0.05)
All	0.27(0.03)	0.34(0.03)	0.33(0.03)	0.34(0.03)
