# Supplementary material for: Incorporating Genome-Wide Association Mapping Results Into Genomic Prediction Models for Grain Yield and Yield Stability in CIMMYT Spring Bread Wheat
Source: Front Plant Sci. 2020 Mar 4;11:197. doi: 10.3389/fpls.2020.00197 (PMC7064468; doi:10.3389/fpls.2020.00197)
Supplement: Supplementary file 1 [file Data_Sheet_1.zip › Table S3.pdf]

S3 Table Summary of haplotype blocks (HBs) on all chromosomes

Chromosome	Number of HBs	Total number of haplotypes	Number of haplotypes in a HB (min -max)	Size of haplotypes in HBs (in bp; min – max)
1A	18	49	2-4	2-5
1B	26	67	2-4	2-6
1D	6	12	2	2
2A	50	130	2-5	2-6
2B	56	148	2-6	2-7
2D	8	16	2	2-4
3A	25	61	2-4	2-5
3B	34	86	2-4	2-9
3D	3	6	2	2-6
4A	23	62	2-5	2-4
4B	11	27	2-5	2-4
4D	1	3	2	2
5A	15	34	2-4	2-8
5B	50	113	2-4	2-7
5D	6	14	2-3	2-5
6A	21	53	2-3	2-4
6B	48	118	2-4	2-7
6D	4	11	2-4	2-4
7A	45	117	2-5	2-7
7B	43	97	2-4	2-6
7D	8	16	2	2-4
