# Supplementary material for: Incorporating Genome-Wide Association Mapping Results Into Genomic Prediction Models for Grain Yield and Yield Stability in CIMMYT Spring Bread Wheat
Source: Front Plant Sci. 2020 Mar 4;11:197. doi: 10.3389/fpls.2020.00197 (PMC7064468; doi:10.3389/fpls.2020.00197)
Supplement: Supplementary file 1 [file Data_Sheet_1.zip › Table S7.pdf]

S7 Table Common markers (green highlighted) identified by Haplotypes- and single SNPs-based GWAS for different environments

| | Haplotypes-based GWAM | Single SNP GWAM | |
|---|---|---|---|
| *B-5IR* | Markers in HBB | *B-5IR* | |
| 1A (HBB1.10) | S1A_535441795 | 1A | S1A_535441795 |
| | S1A_535441797 | | S1A_535441797 |
| 2B (HB5.11, HB5.19, HB5.21) | | 2B | S2B_142247929 |
| HB5.19 | S2B_142247929 | | S2B_142247930 |
| | S2B_142247930 | 7A | S7A_36087756 |
| 7A (HB19.8) | S7A_36087756 | 7B | S7B_57635509 |
| | S7A_36087757 | | |
| | S7A_36096388 | | |
| *B-2IR* | | *B-2IR* | |
| 2B (HB5.1, HB5.53) | | 1B | S1B_637821116 |
| HB5.53 | S2B_795302376 | 2B | S2B_795302376 |
| | S2B_795302390 | | S2B_795302390 |
| 5B (HB14.16) | S5B_387952822 | 5B | S5B_387952822 |
| | S5B_387952832 | | S5B_387952832 |
| | | 6B | S6B_476199639 |
| *SD* | | *SD* | |
| 3A (HB7.4) | | 3B | S3B_40289701 |
| 3B (HB8.6) | S3B_14314433 | 4B | S4B_663459971 |
| | S3B_14314439 | | |
| 4B (HB11.11) | S4B_664556294 | | |
| | S4B_664556306 | | |
| 5B (HB14.49) | | | |
| *B-2IR and SD* | | *B-2IR and SD* | |
| 6B (HBB17.1) | S6B_3567046 | 2A | S2A_211705334 |
| | S6B_3567059 | | S2A_211705336 |
| | S6B_3567083 | 3B | S3B_7031744 |
| | | 6B | S6B_3567046 |
| | | | S6B_3567059 |
| *HS* | | *HS* | |
| 2B (HB5.42) | | 1B | |
| 3B (HB8.26) | | 7B | S7B_713286031 |
| 7B (HB20.38) | S7B_714049759 | | S7B_712486651 |
| | S7B_714050254 | | S7B_614292314 |
| | S7B_714050329 | | |
