# Supplementary material for: Incorporating Genome-Wide Association Mapping Results Into Genomic Prediction Models for Grain Yield and Yield Stability in CIMMYT Spring Bread Wheat
Source: Front Plant Sci. 2020 Mar 4;11:197. doi: 10.3389/fpls.2020.00197 (PMC7064468; doi:10.3389/fpls.2020.00197)
Supplement: Supplementary file 1 [file Data_Sheet_1.zip › Table S9.pdf]

S9 Table Epistatic interactions among main effect loci for GY in different environments

Trial	GY_B-5IR<br>Interacting alleles	R^2 (%)	GY_F-5IR<br>Interacting alleles	R^2 (%)	GY_B-2IR<br>Interacting alleles	R^2 (%)	SD<br>Interacting alleles	R^2 (%)	HS<br>Interacting alleles	R^2 (%)
EYT2011-12	H5.21(CG)<br>H1.10(AG)	6.0	H8.9(AT)<br>H8.2(CC)	6.0	H2.23(AG)<br>H14.16(AC)	3.9	H14.49(CT)<br>H1.11(AA)	5.7	H21.2(CG)<br>H20.1(GT)	4.2
	H5.19(GT)<br>H1.10(AG)	7.3	H11.3(CG)<br>H8.9(AT)<br>H8.2(CC)	6.9	H2.23(AG)<br>H14.16(TT)	3.9	H14.38(GT)<br>H1.11(AA)	5.4	H21.2(CG)<br>H14.38(GT)	4.2
	H5.11(CC)<br>H2.5(AC)	5.7			H19.39(GT)<br>H14.16(AC)	3.7	H11.5(GT)<br>H8.9(AT)	4.5	H21.2(CG)<br>H1.11(AA)	4.3
	H5.21(CG)<br>H4.50(GG)<br>H1.10(AG)	2.1			H4.30(TT)<br>H2.23(AG)<br>H14.16(TT)	4.8	H14.49(CT)<br>H14.38(GT)<br>H1.11(AA)	7.1	H21.2(CG)<br>H20.1(GT)<br>H1.11(AA)	5.9
					H2.23(AG)<br>H19.39(GT)<br>H14.16(TT)	4.8	H14.38(GT)<br>H11.5(GT)<br>H8.9(AT)	6.3		
EYT2012-13	H5.21(CG)<br>H4.50(AA)	0.3	H20.12(AT)<br>H19.1(RY)<br>H2.16(CG)	3.6	H8.28(AC)<br>H19.39(GT)	0.3	H14.49(AT)<br>H1.11(AA)	6.5	H14.38(GT)<br>H8.32(AA)	4.2
			H19.1(RY)<br>H17.36(AG)<br>H4.5(AG)	3.3	H8.28(AC)<br>H4.30(TT)<br>H19.39(GT)	0.5	H14.49(AT)<br>H11.3(CG)<br>H1.11(AA)	6.4		
			H19.1(RY)<br>H11.3(CG)<br>H2.16(CG)	3.3			H14.49(AT)<br>H4.4(CT)<br>H1.11(AA)	7.0		

Trial	GY_B-5IR<br>Interacting alleles	R^2 (%)	GY_F-5IR<br>Interacting alleles	R^2 (%)	GY_B-2IR<br>Interacting alleles	R^2 (%)	SD<br>Interacting alleles	R^2 (%)	HS<br>Interacting alleles	R^2 (%)
EYT2012-13			H19.1(RY)<br>H8.2(CC)<br>H2.16(CG)							
EYT2013-14	H5.21(CT)<br>H4.50(GG)	4.0	H19.1(RY)<br>H7.18(AG)	3.7	H8.2(TT)<br>H7.19(CG)	4.3	H14.38(AG)<br>H8.6(AC)	4.3	H19.1(CG)<br>H17.36(AG)	3.9
	H4.50(GG)<br>H14.38(GT)	3.1	H8.2(TT)<br>H7.19(CG)<br>H4.7(AC)	3.0	H5.53(CC)<br>H4.30(TT)	4.2	H11.3(NN)<br>H8.6(AC)	4.1	H21.2(CG)<br>H19.1(CG)<br>H17.36(AG)	4.3
	H5.21(CT)<br>H4.50(GG)<br>H14.38(GT)	5.3			H7.19(CG)<br>H5.53(AT)<br>H19.39(GT)	1.2	H14.38(AG)<br>H11.4(NN)<br>H8.6(AC)	4.4		
							H14.38(AG)<br>H8.6(AC)<br>H2.16(NN)	4.1		
EYT2014-15	H5.19(GT)<br>H4.50(AA)	3.3			H8.28(TT)<br>H5.1(GT)	0.5	H4.42(TT)<br>H1.11(AA)	3.9	H19.1(AT)<br>H5.42(AC)	3.8
	H5.19(GT)<br>H20.12(AT)	4.2			H8.28(TT)<br>H5.1(GT)<br>H14.16(TT)	0.8	H11.11(AC)<br>H7.18(AG)<br>H1.11(AA)	3.2	H8.32(AA)<br>H4.5(GT)	3.6
	H5.11(CC)<br>H14.38(GT)	4.9	H20.14(GG)<br>H4.38(AA)	3.3					H8.9(AT)<br>H4.5(GT)	3.4
	H5.19(AC)<br>H5.11(GT)<br>H20.12(CG)	9.5	H20.1(CC)<br>H17.36(AG)	3.4						

Trial	GY_B-5IR<br>Interacting alleles	R^2 (%)	GY_F-5IR<br>Interacting alleles	R^2 (%)	GY_B-2IR<br>Interacting alleles	R^2 (%)	SD<br>Interacting alleles	R^2 (%)	HS<br>Interacting alleles	R^2 (%)
EYT2014-15			H20.1(CC)<br>H4.12(TT)	3.4					H11.4(GT)<br>H8.32(AA)<br>H4.12(CC)	6.1
			H20.1(CC)<br>H8.32(AA)<br>H8.9(AT)	3.6						
			H20.1(CC)<br>H17.36(AG)<br>H4.4(CT)	3.7						
EYT2015-16	H5.21(CT)<br>H20.12(CG)	3.4	H21.2(AT)<br>H2.17(GG)	4.1	H5.53(AT)<br>H19.39(CT)	3.5	H14.49(CT)<br>H7.4(GT)	3.6	H19.39(GT)<br>H4.16(CC)	4.5
	H11.3(CG)<br>H5.21(CT)<br>H5.11(CC)	10.1	H21.2(CG)<br>H4.12(TT)	4.0	H7.19(CG)<br>H19.39(CT)	1.7	H11.4(GT)<br>H7.4(GT)	2.9	14.38(GT)<br>H4.16(CC)	4.1
	H11.3(CG)<br>H5.21(CT)<br>H20.12(CG)	8.9	H21.2(CG)<br>H4.7(TT)	3.6	H8.28(AC)<br>H5.53(AT)<br>H19.39(CT)	6.3	H11.4(GT)<br>H7.4(GT)<br>H4.4(CT)	3.4		
			H21.2(AT)<br>H17.36(AG)<br>H2.17(GG)	4.1	H7.19(CG)<br>H5.53(AT)<br>H19.39(CT)	5.3				
			H21.2(CG)<br>H8.28(AC)<br>H4.12(TT)	3.9						
			H11.3(CG)<br>H8.28(AC)<br>H4.8(AT)	3.9						
